# Supplementary figures and images for: A dual-strategy expression screen for candidate connectivity labels in the developing thalamus
Source: PLoS One. 2017 May 30;12(5):e0177977. doi: 10.1371/journal.pone.0177977 (PMC5448750; doi:10.1371/journal.pone.0177977)

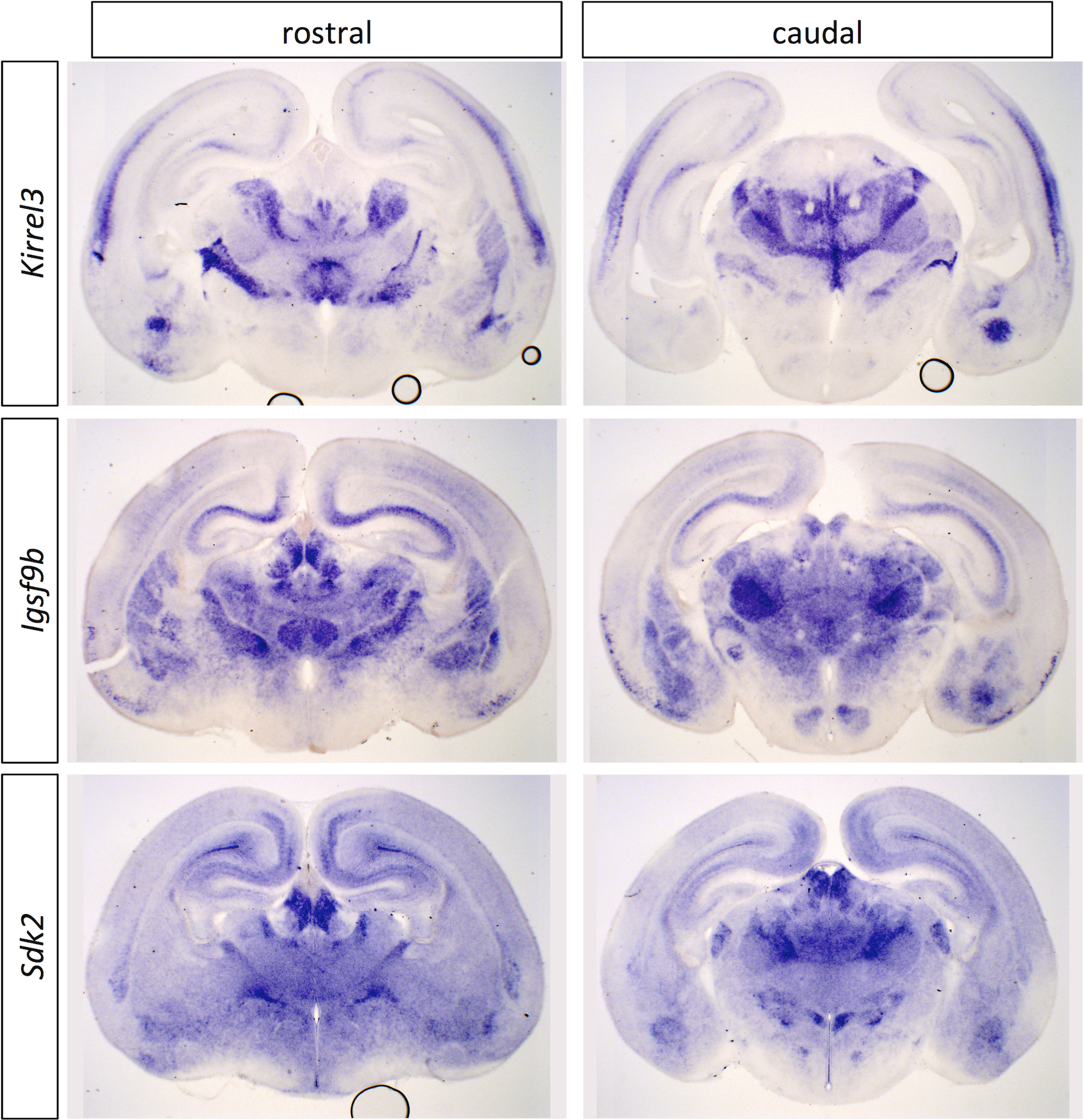

Supplement: S1 Fig — Two coronal sections are shown for Kirrel3, Igsf9b and Sdk2, one rostral and one more caudal. Scale bar: 1 mm. (TIFF) [file pone.0177977.s007.tiff]

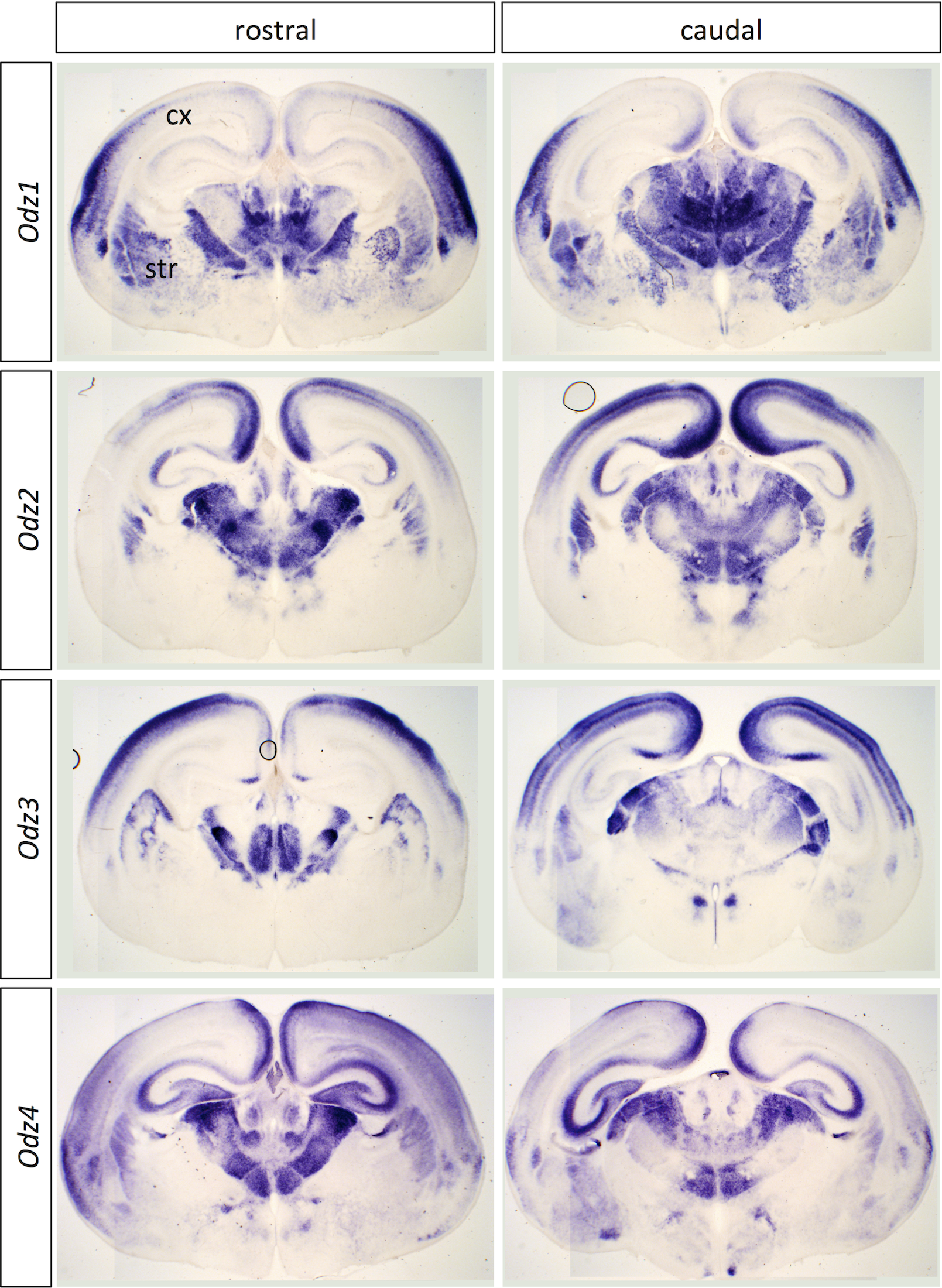

Supplement: S2 Fig — Two coronal sections are shown for Odz1, Odz2, Odz3 and Odz4, one rostral and one more caudal. In addition to restricted expression in dorsal thalamus, there is also graded expression of Odz genes across cortex (cx) and striatum (str) in differing patterns. Scale bar: 1 mm. (TIFF) [file pone.0177977.s008.tiff]

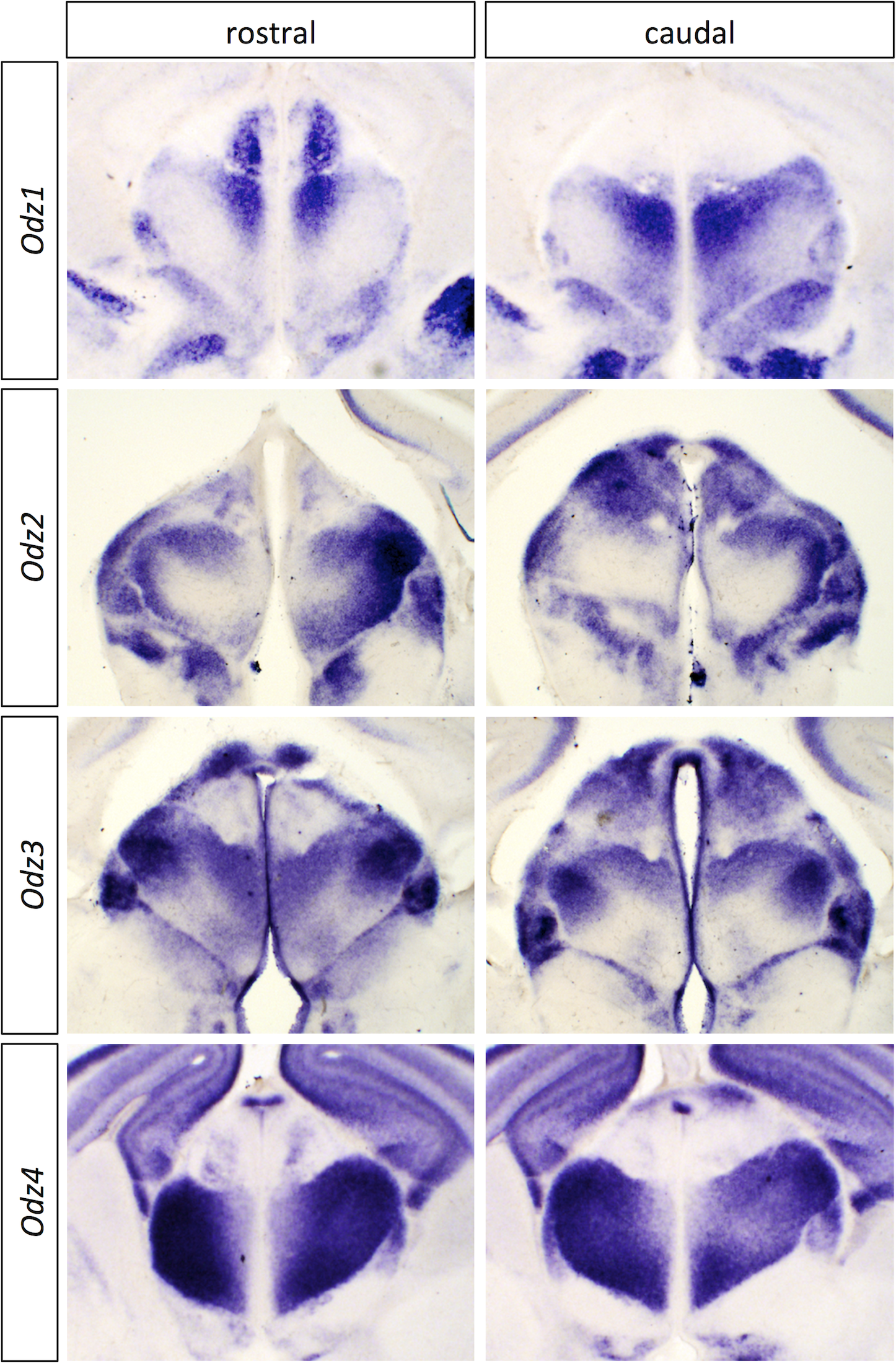

Supplement: S3 Fig — Two coronal sections are shown for Odz1, Odz2, Odz3 and Odz4, one rostral and one more caudal. Differential expression across the dorsal thalamus is already evident at this stage. The corresponding entire brain sections are shown in Figure S4. Scale bar: 500 μm. (TIFF) [file pone.0177977.s009.tiff]

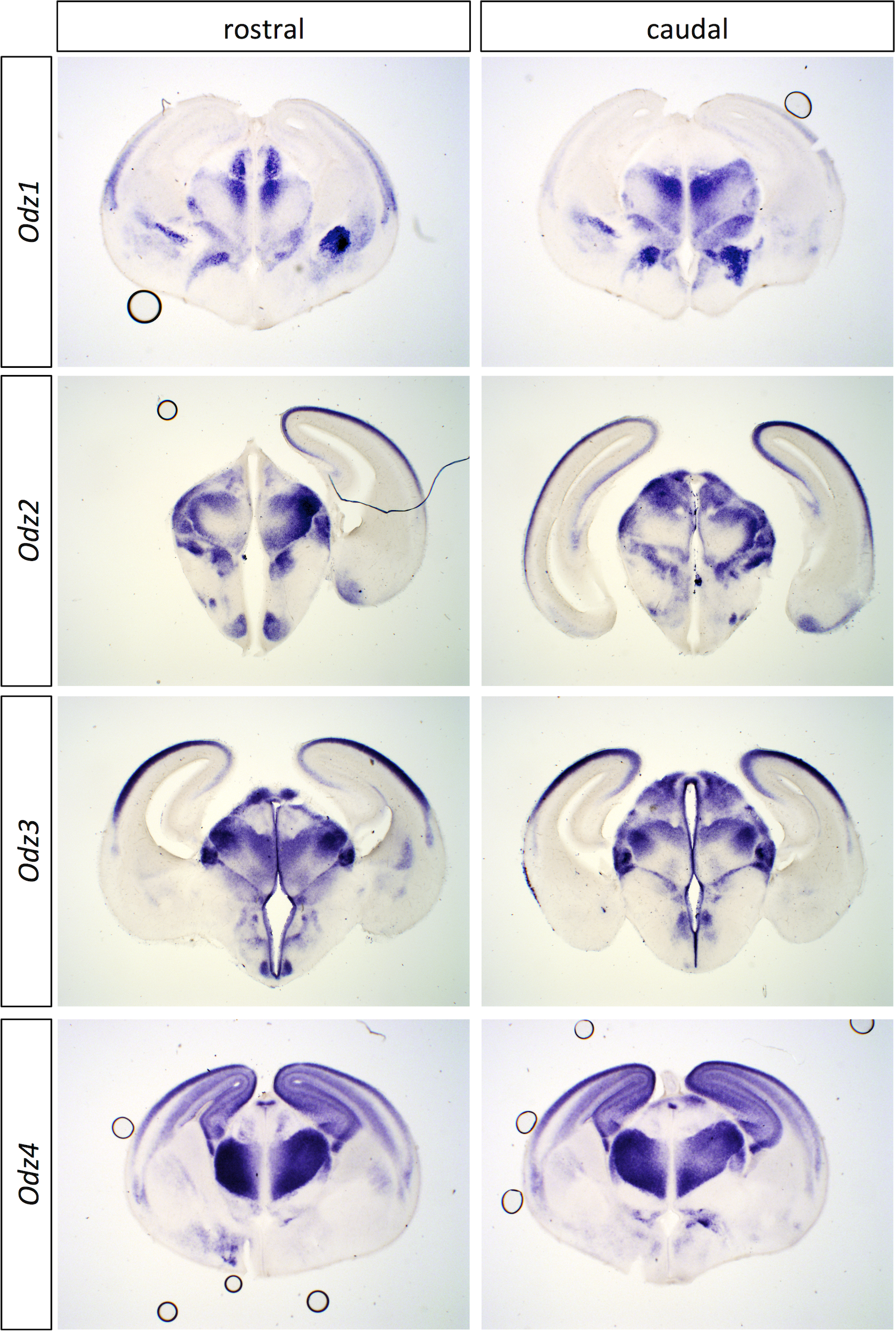

Supplement: S4 Fig — Two coronal sections are shown for Odz1, Odz2, Odz3 and Odz4, one rostral and one more caudal. Scale bar: 1 mm. (TIFF) [file pone.0177977.s010.tiff]

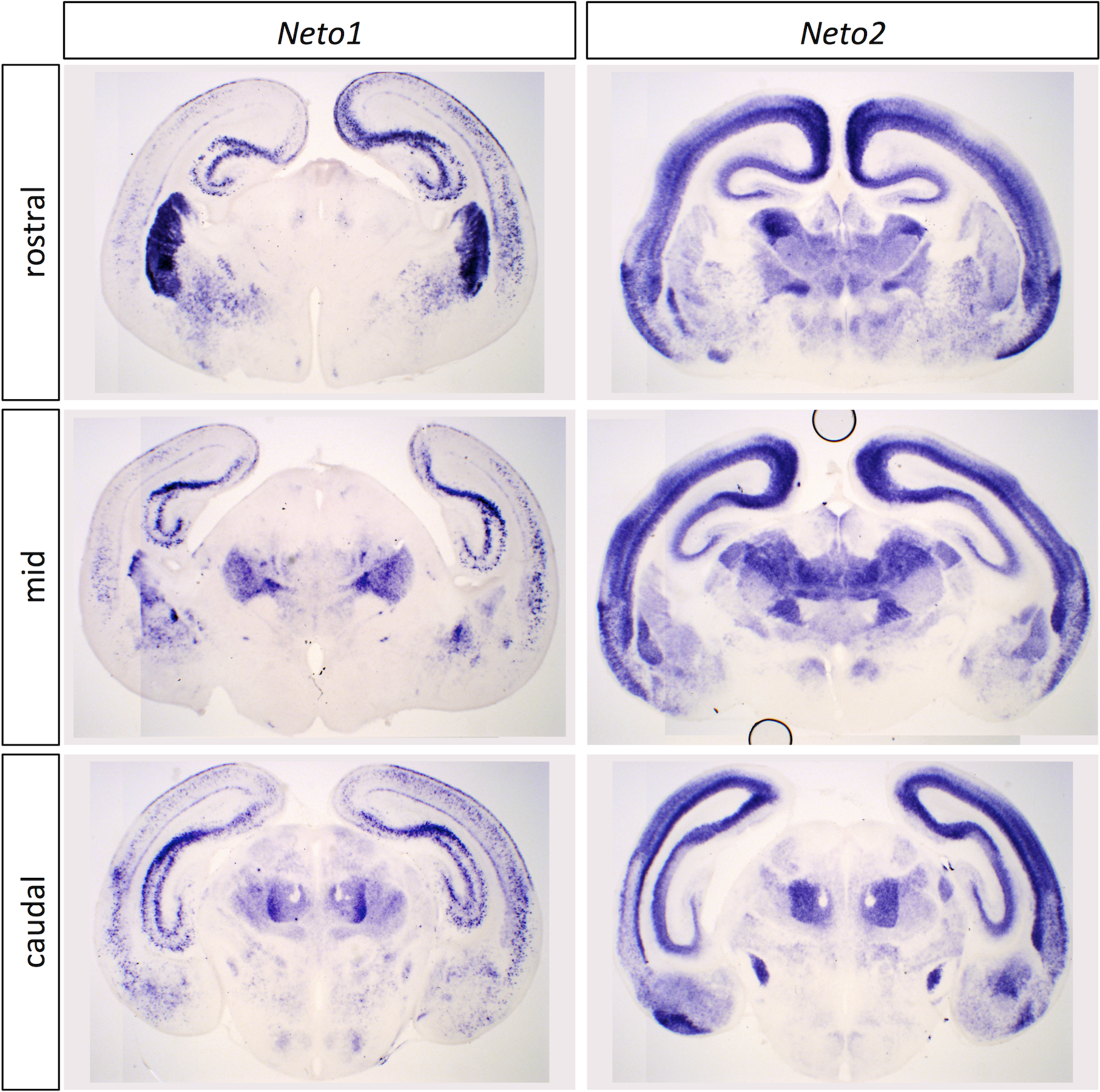

Supplement: S5 Fig — Three coronal sections are shown for Neto1 and Neto2, one rostral, one at an intermediate level (mid) and one more caudal. Scale bar: 1 mm. (TIFF) [file pone.0177977.s011.tiff]

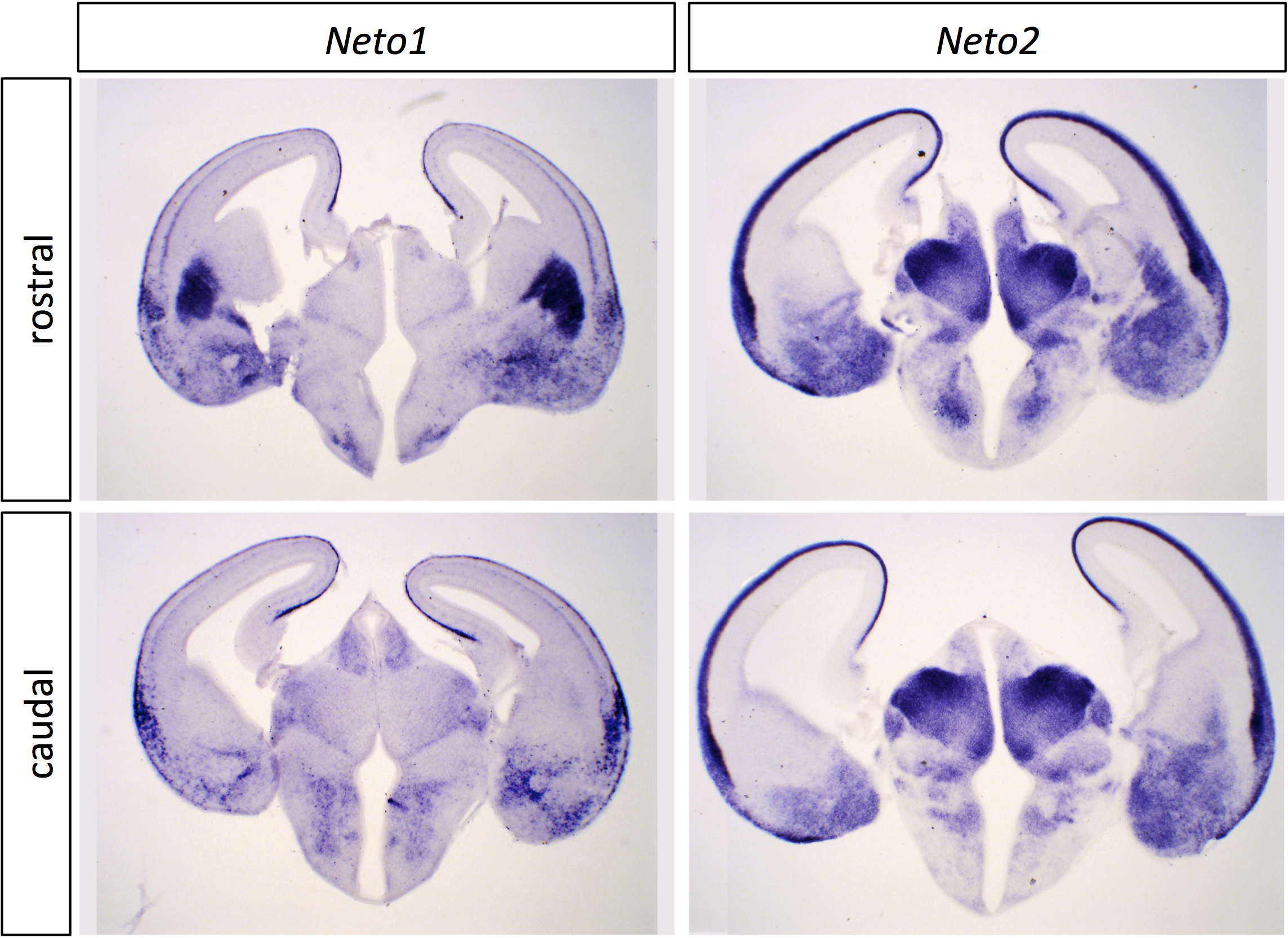

Supplement: S6 Fig — Two coronal sections are shown for Neto1 and Neto2, one rostral and one more caudal. Differential expression across the dorsal thalamus is already evident at this stage. Scale bar: 1 mm. (TIFF) [file pone.0177977.s012.tiff]

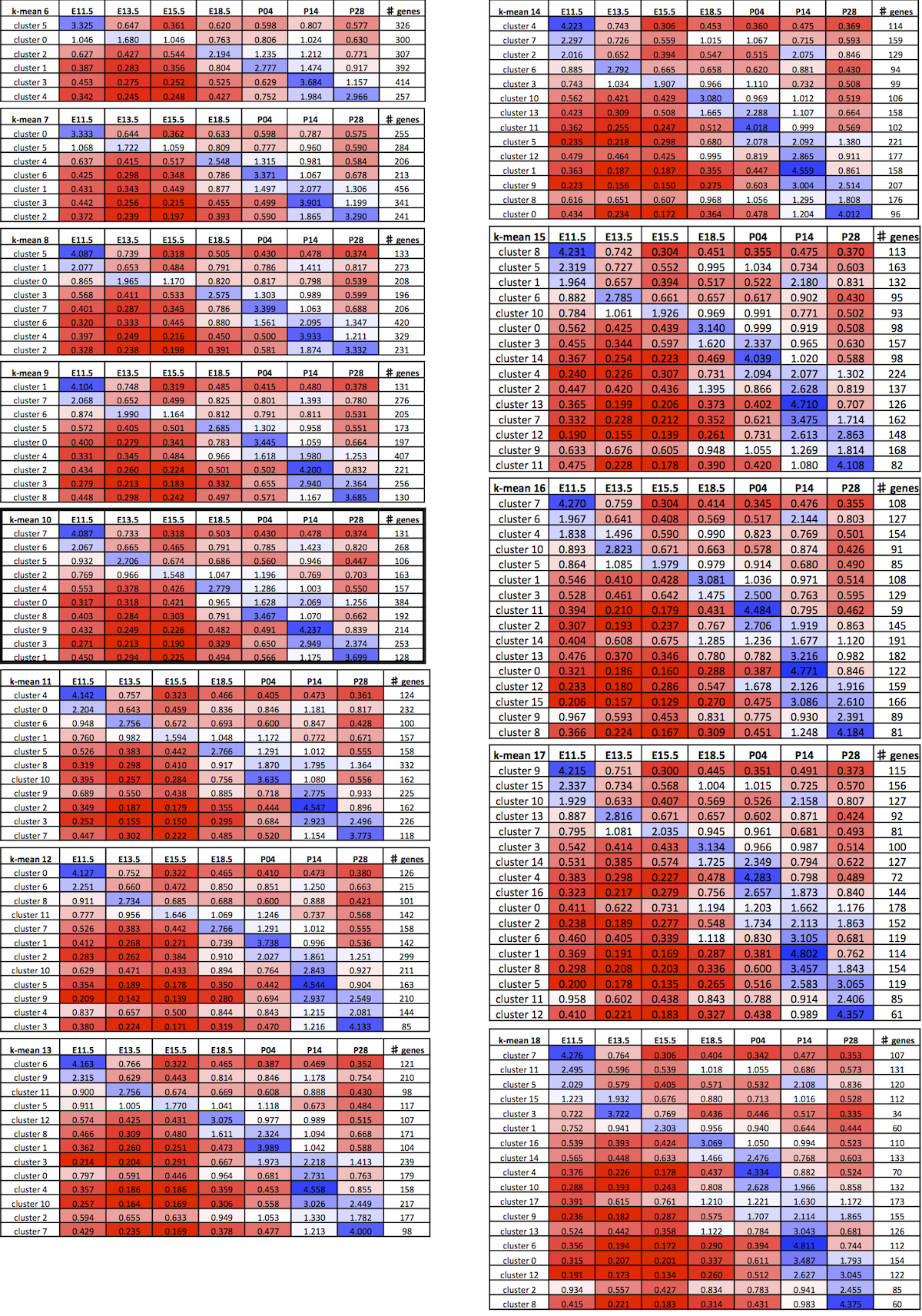

Supplement: S7 Fig — Normalised expression densities were averaged per cluster to see the trends of expression for the results of all clustering analyses from k = 6 to 18 (k indicated at top left corner of each table). Clusters were organised chronologically with early peaks of expression at the top and later peaks of expression at the bottom. Heatmap’s 3 colour scale of gene expression data: 0.2, red; 1, white; 5, blue. k = 10 was used for further analyses; the corresponding tableframe is in bold. (TIF) [file pone.0177977.s013.tif]

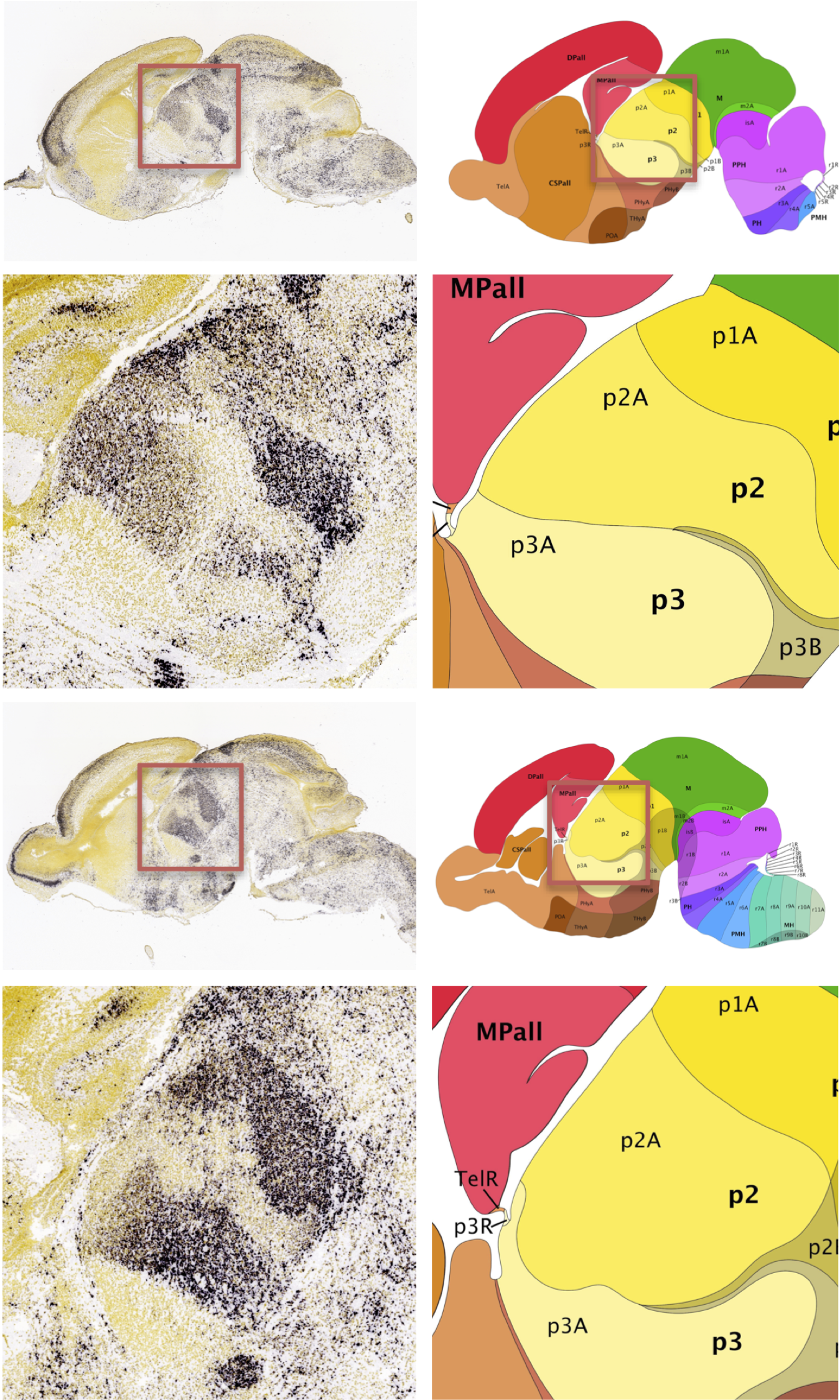

Supplement: S8 Fig — In situ hybridization data from a sagittal section of the thalamus at E18.5 obtained from the devABA (A and E). Corresponding section from the anatomic reference atlas (B and F, respectively). Higher magnifications of the thalamus from squared regions in A, B, E and F, respectively (C, D, G and H). Scale bar in A, B, E and F, 880 μm; C, D, G and H, 214 μm. PT (pretectum) and prethalamus (PTh) are labeled within their region bordering the thalamus. p1, prosomere 1 (pretectum and pretectal tegmentum); p1A, alar plate of prosomere 1; p1B, basal plate of prosomere 1; p2, prosomere 2 (thalamus and thalamic tegmentum); p2A, alar plate of prosomere 2; p2B, basal plate of prosomere 2; p3, prosomere 3 (prethalamus and prethalamic tegmentum); p3A, alar plate of prosomere 3; p3B, basal plate of prosomere 3. Note that voxels were assigned regional labels for thalamus and thalamic tegmentum separately. (TIFF) [file pone.0177977.s014.tiff]

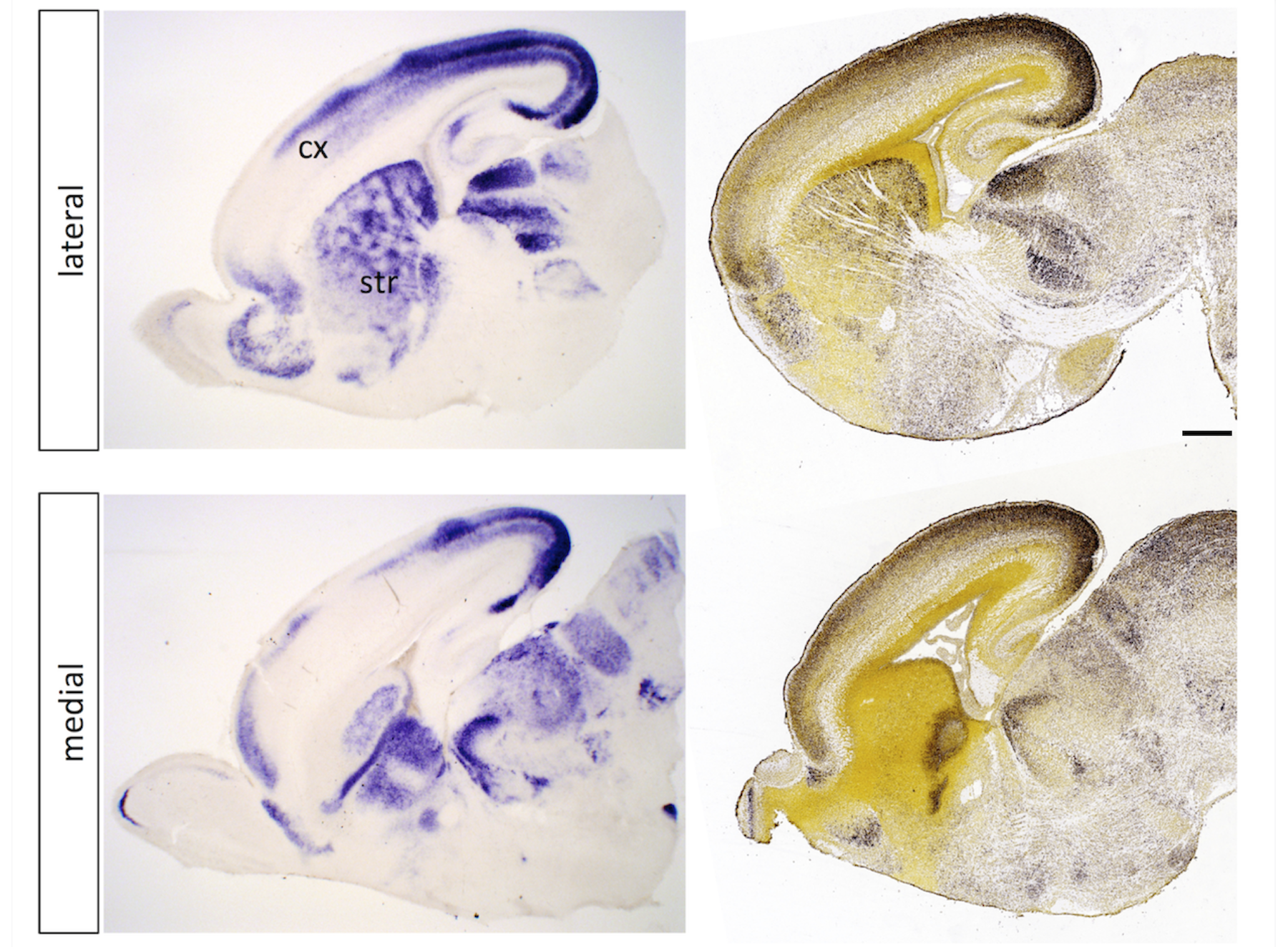

Supplement: S9 Fig — Two sagittal sections are shown, one lateral and one more medial. Our in situ hybridizations are on sections from P0 nenonates, while the devABA sections are from E18.5 embryos. Despite this difference of about a day, there is strikingly good correspondence in expression patterns across the dorsal thalamus, striatum (str), cortex (cx) and other brain regions. Scale bar: 1 mm. (TIFF) [file pone.0177977.s015.tiff]
